# Increased Circulating Angiopoietin-Like Protein 8 Levels Are Associated with Thoracic Aortic Dissection and Higher Inflammatory Conditions

**DOI:** 10.1007/s10557-019-06924-7

**Published:** 2020-02-07

**Authors:** Yunyun Yang, Xiaolu Jiao, Linyi Li, Chaowei Hu, Xiaoping Zhang, Lili Pan, Huahui Yu, Juan Li, Dong Chen, Jie Du, Yanwen Qin

**Affiliations:** 1 Key Laboratory of Remodeling-related Cardiovascular Diseases, Beijing An Zhen Hospital, Beijing Institute of Heart, Lung and Blood Vessel Diseases, Capital Medical University, 2 Anzhen Road, Chaoyang District, Beijing, 100029 China; 2 Key Laboratory of Upper Airway Dysfunction-related Cardiovascular Diseases, Beijing An Zhen Hospital, Beijing Institute of Heart, Lung and Blood Vessel Diseases, Capital Medical University, Beijing, 100029 China; 3 Department of Pathology, Beijing An Zhen Hospital, Capital Medical University, Beijing, 100029 China

**Keywords:** Thoracic aortic dissection, Angiopoietin-like protein 8, Inflammation

## Abstract

**Purpose:**

Thoracic aortic dissection (TAD) is characterized by an inflammatory response. Angiopoietin-like protein 8 (ANGPTL8) is a hormone involved in the regulation of lipid metabolism and inflammation. However, the relationship between ANGPTL8 and TAD remains unknown.

**Methods:**

This case-control study included 78 TAD patients and 72 controls. The aortic diameter was evaluated by computed tomography and used to assess TAD severity. Circulating ANGPTL8 levels were measured by enzyme-linked immunosorbent assay. Associations of ANGPTL8 with TAD were determined by multivariate logistic regression.

**Results:**

Serum ANGPTL8 levels were significantly higher in TAD patients compared with controls (562.50 ± 20.84 vs. 419.70 ± 22.65 pg/mL, respectively; *P* < 0.001). After adjusting for confounding factors, circulating ANGPTL8 levels were an independent risk factor for TAD (odds ratio = 1.587/100 pg ANGPTL8, 95% confidence interval [CI] = 1.121–2.247, *P* < 0.001) and positively associated with diameter (*β* = 1.081/100 pg ANGPTL8, 95% CI = 0.075–2.086, *P* = 0.035) and high-sensitivity C-reactive protein (hs-CRP) (*β* = 0.845/100 pg ANGPTL8, 95% CI = 0.020–1.480, *P* = 0.009). The area under the curve (AUC) on receiver operating characteristic (ROC) analysis of the combination of ANGPTL8, hs-CRP, and D-dimer was 0.927, and the specificity and sensitivity were 98.46% and 79.49%, respectively. ANGPTL8 was significantly increased in TAD tissue compared with controls. In vitro, ANGPTL8 was increased in angiotensin II (AngII)-treated macrophages and vascular smooth muscle cells (VSMCs), while ANGPTL8 siRNA-mediated knockdown decreased inflammatory factors in AngII-treated macrophages and decreased apoptosis in AngII-treated VSMCs.

**Conclusion:**

ANGPTL8 is associated with TAD occurrence and development, which may involve pro-inflammatory effects on macrophages. ANGPTL8 combined with D-dimer and hs-CRP might be a useful clinical predictor of TAD.

**Trial Registration:**

ChiCTR-COC-17010792 http://www.chictr.org.cn/showproj.aspx?proj=18288

**Electronic supplementary material:**

The online version of this article (10.1007/s10557-019-06924-7) contains supplementary material, which is available to authorized users.

## Introduction

Acute thoracic aortic dissection (TAD) is a severe cardiovascular disease with characteristics including acute onset, rapid development, and high morbidity and mortality [1]. TAD results in approximately 10,000 deaths in the USA annually [2]. Many studies have shown a close association of an inflammatory response with the onset and progression of TAD [3]. Local chronic inflammation causing aortic medial degeneration is involved in the pathophysiology of TAD [4]. Indeed, high-sensitivity C-reactive protein (hs-CRP) is used to predict in-hospital events in patients with acute aortic dissection [5]. However, CRP has been preliminarily shown to have less predictive value for aortic disease [6].

There is increasing evidence for a role of the perivascular adipose tissue that surrounds the vasculature in controlling vascular tone and inflammation through local release of adipokines, which contributes to vascular dysfunction [7]. For example, the pleiotropic adipokine adiponectin was reported to inhibit the expression of inflammatory cytokines and mast cell protease in angiotensin II (AngII)-induced abdominal aortic aneurysm [8]. Angiopoietin-like protein 8 (ANGPTL8) is an adipokine and circulating ANGPTL8 is related to metabolic and inflammatory parameters, and oxidative stress, in patients with type 2 diabetes mellitus [9]. ANGPTL8 was also reported to be a novel regulator of inflammation via selective autophagic degradation of Ikappa B kinase IKKγ [10]. Furthermore, ANGPTL2 was abundantly expressed in infiltrating macrophages within the vessel walls of patients with abdominal aortic aneurysms and in a CaCl_2_-induced abdominal aortic aneurysm mouse model, while ANGPTL2-deficient mice showed decreased abdominal aortic aneurysm development compared with wild-type mice and lower expression of pro-inflammatory cytokines [11]. However, the associations of ANGPTL8 and TAD, and the role of ANGPTL8 in TAD development, are unknown. Thus, the aim of the present study was to investigate the association of serum ANGPTL8 levels with the occurrence and severity of TAD, and the potential underling mechanisms.

## Materials and Methods

### Patient Recruitment and Specimens

This case-control study was conducted between August 2018 and January 2019 at the Beijing An Zhen Hospital. A final 150 subjects were recruited into this study, including 78 patients with TAD and 72 controls. The study design is described in detail in Supplemental Figure S1. All participants gave written informed consent before enrollment. The protocol was approved by the Medicine Ethics Committee of Beijing An Zhen Hospital and adhered to the Declaration of Helsinki. This study was registered in the Chinese Clinical Trial Registry (no. ChiCTR-COC-17010792).

A total of 162 TAD patients were consecutively enrolled in this study in the Vascular Department of Beijing An Zhen Hospital. The criteria for diagnosis of TAD were according to the latest diagnostic standard [12]. Patients undergoing conventional open-heart surgery for TAD after pre-operative computed tomography (CT) were included in this study [13]. The maximum axial aortic diameter in the ascending aorta was assessed by CT at the time of presentation in TAD patients [14, 15]. Exclusion criteria included the presence of inherited connective tissue disorders (for example, Marfan syndrome and Ehlers-Danlos syndrome), patients with aortic trauma, pseudo-aneurysm, history of heart failure, renal dysfunction, severe pulmonary diseases, active cancer, previous aortic surgery, or previous treatment with non-steroidal anti-inflammatory drugs or steroids [14, 16]. Finally, we enrolled 78 patients with TAD.

A total of 162 volunteers from the Health Examination Center at Beijing An Zhen Hospital were enrolled as controls. Control patients were enrolled only if the CT revealed no evidence of aortic diseases. Maximum axial aortic diameters in the ascending aorta were assessed by CT [13]. Exclusion criteria were the same as the TAD cases. The presence of risk factors in patients, including hypertension, dyslipidemia, diabetes, coronary artery disease, and tobacco and alcohol use, were collected from medical records, as previously described [17]. We enrolled 72 controls, who were matched for sex, age, and body mass index (BMI). No sex-based or race/ethnicity-based differences were present.

TAD tissues were dissected during surgery. Control aortic samples were obtained from heart transplantation donors in Beijing An Zhen Hospital, as previously described [18], while donors with collagen disease or aortic aneurysm/dissection were excluded. Tissue specimens were fixed in 10% formalin, embedded in paraffin, and sectioned at 5 μm thickness (*n* = 6 per group).

### Diagnostic Criteria

Demographic data, risk factors, and laboratory parameters were obtained from clinical records. Hypertension was defined as systolic blood pressure (SBP) ≥ 140 mmHg, and/or diastolic blood pressure (DBP) ≥ 90 mmHg, and/or being under antihypertensive treatment [19]. Diabetes was defined as fasting serum glucose (FPG) ≥ 7 mmol/L (126 mg/dL) and/or being on treatment for diabetes [20]. At least one of the following was present in patients with dyslipidemia: high triglyceride (TG), high cholesterol (TC), high low-density lipoprotein cholesterol (LDL-C), high non-high-density lipoprotein cholesterol (non-HDL-C), and low HDL-C levels. High serum TG level was defined as a serum TG ≥ 1.7 mmol/L. High serum TC level was defined as a serum TC ≥ 5.2 mmol/L. High serum LDL-C level was defined as a serum LDL-C ≥ 3.4 mmol/L. High serum non-HDL-C level was defined as a serum non-HDL-C ≥ 4.1 mmol/L. Low serum HDL-C level was defined as a serum HDL-C < 1.0 mmol/L [21].

### Anthropometric Measurements

Anthropometric determinations and blood extractions were performed on a single day. Height and weight were measured with participants wearing light indoor clothing and barefoot using calibrated portable electronic weighing scales and portable inflexible height measuring bars. Blood pressure was measured after a 5-min rest in a seated position. Blood pressure was determined at least three times on the right upper arm, and the mean value was used for final analyses. BMI was calculated using the standard BMI formula: body mass (kg) divided by square of height (m^2^).

### Blood Sample Preparation

Blood samples were collected after the participants had fasted overnight. Samples were centrifuged for 10 min at 3000×*g* at 4 °C, and plasma samples were stored at − 80 °C before analysis. Serum TG, TC, LDL-C, and HDL-C levels, along with other routine serum biochemical parameters, were measured in a biochemical analyzer (Hitachi-7600; Hitachi, Tokyo, Japan) using blinded quality control specimens in the Department of the Biochemical Laboratory at Beijing An Zhen Hospital. Circulating full-length ANGPTL8 levels were measured using an enzyme-linked immunosorbent assay kit (#11644h; Wuhan ELAAB Science, Wuhan, China) according to the manufacturer’s instructions. Intra- and inter-assay coefficients of variation for ANGPTL8 levels were < 5% and < 10%, respectively.

### Histology and Immunohistochemistry

Immunohistochemical staining was performed as previously described [22] using primary antibodies against ANGPTL8 (1:200 dilution; Abcam, Cambridge, UK), α-smooth muscle actin (α-SMA) (1:200 dilution; ZM0003, ZSGB-BIO, Beijing, China), or galectin 2 (MAC-2) (1:200 dilution; Abcam, Cambridge, UK), followed by staining with secondary antibodies. Images were obtained with a Ni-UNikon Upright Microscope equipped with a DS-Ri2 color CCD (Nikon, Tokyo, Japan).

### Cell Culture and Conditions

Human umbilical artery smooth muscle cells (HUASMCs; Shanghai Xinyu Biotech Co., China Ltd.) were cultured in low-glucose DMEM medium (Science Cell, CA, USA) supplemented with 10% fetal bovine serum and 1% penicillin/streptomycin. RAW264.7 cells (American Type Culture Collection, Manassas USA) were cultured in DMEM (Science Cell) with 10% fetal bovine serum and 1% penicillin/streptomycin. Cells were divided into four groups: control group, ANGPTL8 small interfering RNA (siRNA) group, AngII group, and AngII + ANGPTL8 siRNA group.

### siRNA Studies

ANGPTL8-specific siRNA and control siRNA were purchased from Life Technologies Co. The ANGPTL8 siRNA sequences were as follows: sense 5′-GAGAAUUUGAGGUCUUAAAtt-3′ and sense 5′-GGAUAUUCUGCAGCUGCAGTT-3′. HUASMCs and RAW264.7 cells were plated in dishes (60 mm) and grown to 40% confluence prior to transfection. Cells were transfected with 25 pmol siRNA using Lipofectamine Plus (Invitrogen, CA, USA) following the manufacturer’s instructions. After culturing for 12 h, cells in the AngII group and AngII + ANGPTL8 siRNA group were treated with AngII at 25, 50, or 100 nmol/L. After culturing for 24 h, the mRNA levels of interleukin (IL-1B, IL-6), tumor necrosis factor (TNF-α), matrix metalloproteinase (MMP9), B cell lymphoma/leukemia-2 (Bcl-2), and Bcl-2-interacting mediator of cell death (Bim) in the RAW264.7 and HUASMC cells of each group were measured.

### Real-Time qPCR

RNA was extracted from thoracic aortic samples using TRIzol, and 1 μg RNA was reverse transcribed using a GoScript™ reverse transcription system (Promega), according to the manufacturer’s instructions. The iQ5 system (Bio-Rad, Hercules, CA, USA) with SYBR Green I (Takara, Shiga, Japan) was used for real-time qPCR. Samples were amplified by incubation at 95 °C for 5 min, followed by 45 cycles of 95 °C for 45 s and 60 °C for 60 s. The housekeeping gene glyceraldehyde 3-phosphate dehydrogenase was used as a control. Relative mRNA levels were calculated using the 2^−ΔΔCt^ method and normalized to glyceraldehyde 3-phosphate dehydrogenase mRNA levels.

### Western Blot

Protein from RAW264.7 and HUASMC cells was extracted using a protein extraction kit containing protease inhibitors and a protein phosphatase inhibitor cocktail. Equal amounts of protein (40 μg/lane) were separated by 15% SDS-PAGE and then transferred onto a PVDF membrane. Blots were probed overnight at 4 °C with anti-β-actin (1:1000; Abcam) or anti-ANGPTL8 (1:1000; Abcam) antibodies, washed with Tris-buffered saline containing Tween 20, and then incubated with secondary antibodies (1:10,000; ZSGB-BIO) for 1 h at room temperature. Blots were then washed, incubated with SuperSignal™ WestFemto Maximum Sensitivity Substrate (Thermo Fisher Scientific, Waltham, MA, USA), and analyzed using a ChemiDoc™ Touch Imaging System (Bio-Rad).

### TUNEL Staining

Terminal deoxynucleotidyl transferase dUTP nick end labeling (TUNEL) staining was performed according to the manufacturer’s instructions (Roche #12156792910). In short, HUASMCs were plated in dishes (60 mm) with slides, and after AngII and/or ANGPT8 siRNA treatment, cells were embedded in paraffin for 30 min and then treated with 0.01% Triton for 2 min on ice. Slides were then treated with TUNEL reaction mixture in a humidified chamber and washed with PBS. Finally, slides were mounted using Vectashield mounting medium with DAPI (ZLI-9557, ZSGB-BIO, Beijing, China). Images were obtained with a Ni-UNikon Upright Microscope equipped with a DS-Ri2 color CCD (Nikon, Tokyo, Japan).

### Enzyme-Linked Immunosorbent Assay

The inflammatory cytokines in the culture supernatant of RAW264.7 cells treated with AngII and ANGPTL8 siRNA were quantified using enzyme-linked immunosorbent assay (ELISA) kits (RayBiotech, Inc. Systems, Minneapolis, USA) following the manufacturer’s instructions, including IL-6, TNF-α, IL-1β and monocyte chemotactic protein-1 (MCP-1) ELISA kits. The absorbance was measured in a microplate reader at 450 nm. The concentrations of IL-6, TNF-α, IL-1β, and MCP-1 were normalized and calculated based on the linear calibration curves obtained by standard solutions.

### Statistical Analysis

Continuous variables are expressed as mean ± standard deviation and categorical variables as numerals (percentages). The independent Student *t* test for normal distribution and the Wilcoxon rank-sum test for asymmetric distribution were used to analyze the differences in continuous variables. The chi-square test was used to analyze categorical variables. The association of circulating full-length ANGPTL8 levels with TAD was determined by multivariate logistic regression analysis. The association of circulating full-length ANGPTL8 levels with hs-CRP was also evaluated using multivariable linear regression analysis. The variables that may influence the expression of ANGPTL8 and the occurrence of TAD were selected for adjustment [16, 23]. A *P* value < 0.05 was considered statistically significant. In addition, receiving operator curves (ROC) were calculated for the prediction of TAD based on ANGPTL8, hs-CRP, and D-dimer levels and the area under the curve (AUC), sensitivity, and specificity. An AUC of 0.5 indicated no predictive power, whereas an AUC of 1 indicated perfect prediction. Statistical analyses were performed with statistical software (SPSS v20.0; IBM Co., Armonk, NY, USA).

## Results

### Baseline Clinical Characteristics of the Study Population

The clinical characteristics of the TAD patients and controls are shown in Table 1. There were no differences in LDL-C (*P* = 0.952), TC (*P* = 0.318), TG (*P* = 0.287), or FPG (*P* = 0.207) levels between the two groups. Patients with TAD had significantly higher SBP (*P* < 0.001), DBP (*P* < 0.015), homocysteine(Hcy; *P* < 0.031), hs-CRP (*P* < 0.001), D-dimer levels (*P* < 0.001), diameter (*P* < 0.001), and lower HDL-C levels (*P* = 0.004), compared with controls. Circulating ANGPTL8 levels were also significantly higher in the TAD group compared with controls (562.50 ± 20.84 pg/mL vs. 419.70 ± 22.65, respectively; *P* < 0.001). Human serum hs-CRP levels were significantly higher in TAD patients compared with controls (10.95 ± 1.24 mmol/L vs. 1.67 ± 0.16, respectively; *P* < 0.001).

**Table 1:** Anthropometric and biochemical characteristics of the subjects included in the study

	Control (*n* = 72)	TAD (*n* = 78)	*P*
Age (years)	53.09 ± 13.29	51.05 ± 12.36	0.342
BMI (kg/m^2^)	25.22 ± 3.59	26.10 ± 5.59	0.276
Male (*n*, %)	50 (69.44%)	60 (76.92%)	0.301
SBP (mmHg)	119.34 ± 10.59	135.77 ± 20.50	< 0.001*
DBP (mmHg)	73.21 ± 8.10	77.36 ± 11.41	0.015*
FPG (mmol/L)	5.17 ± 0.43	5.29 ± 0.67	0.207
TG (mmol/L)	1.17 (0.76–1.90)	1.23 (0.94–1.90)	0.287
TC (mmol/L)	4.30 ± 0.71	4.09 ± 0.89	0.128
LDL-C (mmol/L)	2.57 ± 0.61	2.56 ± 0.77	0.952
HDL-C (mmol/L)	1.15 ± 0.31	1.00 ± 0.26	0.004*
Hypertension	3 (4.17%)	48 (61.54%)	< 0.001**
Diabetes	2 (2.78%)	3 (3.85%)	0.538
Hyperlipidemia	10 (13.89%)	15 (19.23%)	0.256
Smoker (*n*, %)	18 (25.00%)	27 (34.62%)	0.134
Drinker (*n*, %)	22 (30.56%)	20 (25.64%)	0.314
Hcy (μmol/L)	11.10 (9.07–15.53)	14.60 (9.50–18.30)	0.031*
ANGPTL8 (pg/mL)	419.70 ± 22.65	562.50 ± 20.84	< 0.001**
hs-CRP (mmol/L)	1.67 ± 0.16	10.95 ± 1.24	< 0.001**
D-Dimer (μg/mL)	0.07 (0.04–0.11)	0.64 (0.13–1.52)	< 0.001**
Diameter (mm)	30.08 ± 4.88	46.52 ± 11.40	< 0.001**

Date are expressed as mean ± standard deviation, median (interquartile range), or *n* (%). Differences between the groups were analyzed by an independent Student’s *t* test, *χ*^2^ text, or Wilcoxon test

*TAD* thoracic aortic dissection, *BMI* body mass index, *SBP* systolic blood pressure, *DBP* diastolic blood pressure, *FPG* fasting plasma glucose, *TG* triglycerides, *TC* total cholesterol, *LDL-C* low-density lipoprotein cholesterol, *HDL-C* high-density lipoprotein cholesterol, *Hcy* homocysteine

*P < 0.05, **P < 0.001

### Association of Plasma ANGPTL8 with Clinical or Biochemical Variables

As shown in Table 2, Spearman’s correlation analysis indicated that ANGPTL8 levels were associated with Hcy, maximum axial aortic diameter, D-dimer, and hs-CRP. To investigate the effect of TAD and hypertension on ANGPTL8 expression, TAD patients were grouped according to hypertension status. However, ANGPTL8 levels were not significantly different in TAD subjects according to the presence or absence of hypertension (584.95 ± 30.49 pg/mL vs. 548.45 ± 28.05 pg/mL, respectively; *P* = 0.40; Supplemental Figure S2). These results suggest that ANGPTL8 in TAD patients was not affected by hypertension. We also analyzed the association of antihypertensive drugs with serum ANGPTL8 levels (Supplemental Figure S3). However, patients undergoing drug treatment showed no differences in serum ANGPTL8 levels compared with patients without drug treatment.

**Table 2:** Associations of clinical or biochemical variables with ANGPTL8

	Correlation coefficient	*P* value
Age (year)	0.102	0.225
Sex (male = 1, female = 0)	0.060	0.473
BMI (kg/m^2^)	− 0.086	0.304
SBP (mmHg)	0.136	0.105
DBP (mmHg)	0.005	0.949
TG (mmol/L)	− 0.030	0.717
TC (mmol/L)	− 0.140	0.094
LDL-C (mmol/L)	− 0.020	0.815
HDL-C (mmol/L)	− 0.079	0.345
FPG (mmol/L)	0.109	0.193
hs-CRP (mmol/L)	0.302	< 0.001**
Diameter (mm)	0.271	0.023*
Hcy (μmol/L)	0.174	0.038*
D-Dimer (μg/mL)	0.251	0.002*

Spearman correlation test

*BMI* body mass index, *SBP* systolic blood pressure, *DBP* diastolic blood pressure, *FPG* fasting plasma glucose, *TG* triglycerides, *TC* total cholesterol, *LDL-C* low-density lipoprotein cholesterol, *HDL-C* high-density lipoprotein cholesterol, *Hcy* homocysteine, *P < 0.05, **P < 0.001

### Association of Circulating ANGPTL8 Levels with TAD

The association of TAD with circulating full-length ANGPTL8 levels was tested in different models of logistic regression. After adjustment for age, sex, BMI, SBP, DBP, TG, TC, LDL-C, HDL-C, FPG, Hcy, and hs-CRP, increasing circulating ANGPTL8 levels conferred a higher odds ratio (OR) of TAD (OR = 1.587/100 pg ANGPTL8, 95% confidence interval [CI] = 1.121–2.247, *P* = 0.009; Table 3). In addition, we divided the TAD patients into low rupture risk (< 50 mm) and high rupture risk (> 50 mm) groups according to artery diameter. Multivariate logistic regression analysis showed that, after adjustment for age, sex, BMI, SBP, DBP, TG, TC, LDL-C, HDL-C, FPG, Hcy, and hs-CRP, increased circulating ANGPTL8 levels were associated with a higher OR in TAD with high rupture risk (OR = 2.224/100 pg ANGPTL8, 95% CI = 1.274–3.883, *P* = 0.005; Table 4).

**Table 3:** Multivariate logistic regression analyses of circulating full-length ANGPTL8 levels and TAD

	Unadjusted		Model 1		Model 2	
	OR (95% CI)	*P* value	OR (95% CI)	*P* value	OR (95% CI)	*P* value
ANGPTL8 (per 100pg increase)	1.525 (1.246–1.866)	< 0.001**	1.630 (1.297–2.048)	< 0.001**	1.587 (1.121–2.247)	0.009*

Model 1: adjusted for age, sex, and BMI; Model 2: adjusted for Model 1 + SBP, DBP, TG, TC, LDL-C, HDL-C, Hcy, FPG, and hs-CRP

*ANGPTL8* angiopoietin-like protein 8, *TAD* thoracic aortic dissection, *BMI* body mass index, *SBP* systolic blood pressure, *DBP* diastolic blood pressure, *TG* triglycerides, *TC* total cholesterol, *LDL-C* low-density lipoprotein cholesterol, *HDL-C* high-density lipoprotein cholesterol, *Hcy* homocysteine, *FPG* fasting plasma glucose, *hs-CRP* high-sensitivity C-reactive protein

*P < 0.05, **P < 0.001

**Table 4:** Multivariate logistic regression analyses of circulating full-length ANGPTL8 levels and TAD with high rupture risk (diameter > 50 mm)

	Unadjusted		Model 1		Model 2	
	OR (95% CI)	*P* value	OR (95% CI)	*P* value	OR (95% CI)	*P* value
ANGPTL8 (per 100pg increase)	1.431 (1.148–1.784)	0.001*	1.707 (1.290–2.258)	< 0.001**	2.224 (1.274–3.883)	0.005*

Model 1: adjusted for age, sex, and BMI; Model 2: adjusted for Model 1 + SBP, DBP, TG, TC, LDL-C, HDL-C, Hcy, FPG, and hs-CRP

*ANGPTL8* angiopoietin-like protein 8, *TAD* thoracic aortic dissection, *BMI* body mass index, *SBP* systolic blood pressure, *DBP* diastolic blood pressure, *TG* triglycerides, *TC* total cholesterol, *LDL-C* low-density lipoprotein cholesterol, *HDL-C* high-density lipoprotein cholesterol, *Hcy* homocysteine, *FPG* fasting plasma glucose, *hs-CRP* high-sensitivity C-reactive protein

*P < 0.05, **P < 0.001

### Association of Circulating ANGPTL8 Levels with Diameter, hs-CRP

Multivariate linear regression analysis was used to examine the association of circulating ANGPTL8 levels with the maximum axial aortic diameter, hs-CRP. After adjustment for age, sex, BMI, SBP, DBP, FPG, TG, TC, LDL-C, and HDL-C, circulating full-length ANGPTL8 levels were associated with maximum axial aortic diameter (*β* = 1.081/100 pg ANGPTL8, 95% CI = 0.075–2.086, *P* = 0.035, Table 4). After adjusting for age, sex, BMI, SBP, DBP, FPG, TG, TC, LDL-C, HDL-C, and Hcy, circulating full-length ANGPTL8 levels were positively associated with hs-CRP (*β* = 0.845/100 pg ANGPTL8, 95% CI = 0.020–1.480, *P* = 0.009, Table 5). However, ANGPTL8 was not associated with D-dimers (*P* = 0.059). These results suggest that ANGPTL8 may affect the maximum axial aortic diameter and inflammation.

**Table 5:** Multivariate linear regression analyses of circulating full-length ANGPTL8 levels and diameter

	Unadjusted		Model 1		Model 2	
	*Β* (95% CI)	*P* value	*Β* (95% CI)	*P* value	*Β* (95% CI)	*P* value
ANGPTL8 (per 100 pg increase)	1.312 (0.317–2.306)	0.010*	1.280 (0.291–2.268)	0.012*	1.081 (0.075–2.086)	0.035*

Model 1: adjusted for age, sex, and BMI; Model 2: adjusted for Model 1 + TG, TC, LDL-C, HDL-C, and FPG

*ANGPTL8* angiopoietin-like protein 8, *BMI* body mass index, *TG* triglycerides, *TC* total cholesterol, *LDL-C* low-density lipoprotein cholesterol, *HDL-C* high-density lipoprotein cholesterol, *FPG* fasting plasma glucose

*P < 0.05

### ROC Curve Analysis for ANGPTL8, hs-CRP, and D-Dimer

ROC curve analyses were constructed to evaluate the predictive values of plasma ANGPTL8 and hs-CRP levels for the identification of TAD (Table 6). The results of ANGPTL8 showed an AUC of 0.746 (95% CI = 0.663–0.829; *P* < 0.001). The hs-CRP for the identification of TAD with an AUC of 0.807 (95% CI = 0.736–0.878, *P* < 0.001), the AUC of D-dimer was 0.877 (95% CI = 0.821–0.932; *P* < 0.001).

**Table 6:** Multivariate linear regression analyses of circulating full-length ANGPTL8 levels and hs-CRP

	Unadjusted		Model 1		Model 2	
	*Β* (95% CI)	*P* value	*Β* (95% CI)	P value	*Β* (95% CI)	*P* value
ANGPTL8 (per 100 pg increase)	1.211 (0.519–1.904)	0.001*	1.096 (0.440–1.751)	0.001*	0.845 (0.020–1.480)	0.009*

Model 1: adjusted for age, sex, and BMI; Model 2: adjusted for Model 1 + SBP, DBP, TG, TC, LDL-C, HDL-C, and FPG

*ANGPTL8* angiopoietin-like protein 8, *BMI* body mass index, *SBP* systolic blood pressure, *DBP* diastolic blood pressure, *TG* triglycerides, *TC* total cholesterol, *LDL-C* low-density lipoprotein cholesterol, *HDL-C* high-density lipoprotein cholesterol, *FPG* fasting plasma glucose, *Hcy* homocysteine, *hs-CRP* high-sensitivity C-reactive protein

*P < 0.05

### Combination of Plasma ANGPTL8, hs-CRP with D-Dimer

**Fig. 1:**
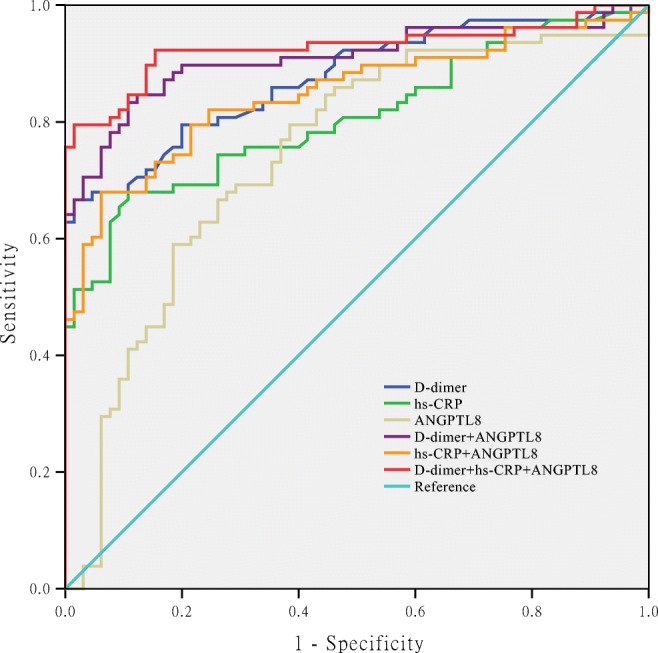
Receiving operator curves using ANGPTL8 and hs-CRP plasma levels for prediction of TAD. The area under the curve (AUC) for the plasma ANGPTL8 levels was 0.746, for hs-CRP levels was 0.807, for D-dimer was 0.877, and for the combination of ANGPTL8, hs-CRP, and D-dimer was 0.927

Next, we analyzed the combined use of plasma ANGPTL8 and D-dimer or hs-CRP. Indeed, calculation of the AUC on ROC analysis demonstrated better performance of a combination of ANGPTL8 and D-dimer than plasma D-dimer alone (AUC 0.909 vs. 0.877); the specificity increased from 66.67% when used alone to 89.39%. A combination of ANGPTL8 and hs-CRP also increased the AUC (0.849 vs. 0.807); the specificity increased from 89.23 to 93.85% (Table 6, Fig. 1). The AUC combination of ANGPTL8, hs-CRP, and D-dimer was up to 0.927 (Table 7), and the sensitivity and specificity were 98.46% and 79.49%, respectively (Table 7).

**Table 7:** Receiving operator curves using ANGPTL8, hs-CRP, and D-dimer levels for prediction of TAD

Parameters	AUC	95% CI	Specificity	Sensitivity	*P*
hs-CRP	0.807	0.736–0.878	0.892	0.679	< 0.001
ANGPTL8	0.746	0.663–0.829	0.621	0.795	< 0.001
D-Dimer	0.877	0.821–0.932	0.667	0.985	< 0.001
hs-CRP + ANGPTL8	0.849	0.784–0.912	0.939	0.679	< 0.001
D-Dimer + ANGPTL8	0.909	0.857–0.959	0.894	0.833	< 0.001
D-Dimer + hs-CRP + ANGPTL8	0.927	0.880–0.974	0.985	0.795	< 0.001

*ANGPTL8* angiopoietin-like protein 8, *TAD* thoracic aortic dissection, *SBP* systolic blood pressure, *DBP* diastolic blood pressure, *hs-CRP* high-sensitivity C-reactive protein

### ANGPTL8 Expression in Human TAD Tissues

**Fig. 2:**
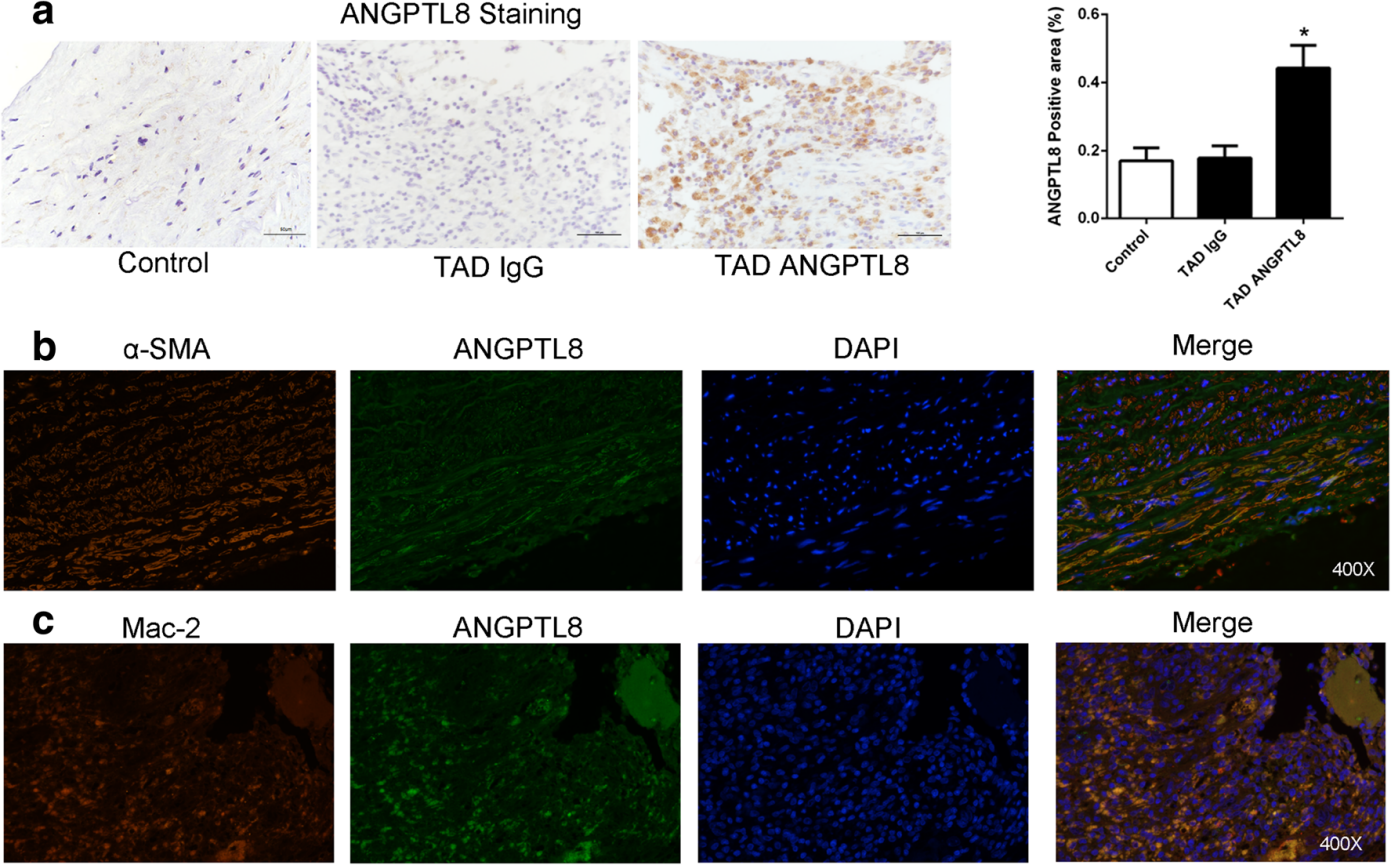
ANGPTL8 is increased in thoracic aortic dissection (TAD) tissues and expressed in macrophages and vascular smooth cells. **a** Representative immunostaining and semiquantitative analysis of ANGPTL8 in the aortas of TAD and control patients (*n* = 6 per group). Data are presented as mean ± standard error of the mean (SEM). *P < 0.05. **b** Co-staining of α-SMA (red) and ANGPTL8 (green) in TAD tissues. **c** Co-staining of MAC-2 (red) and ANGPTL8 (green) in TAD tissues. Data are presented as mean ± SEM. *P < 0.05

Next, we evaluated ANGPTL8 expression in TAD tissues by immunofluorescence staining. ANGPTL8 expression was significantly increased in TAD tissues compared with controls (*P* < 0.05; Fig. 2a). Double immunofluorescence staining showed that vascular smooth muscle cells (VSMCs) and macrophages expressed ANGPTL8 (Fig. 2b, c).

### Effect of ANGPTL8 on Macrophage Inflammation

**Fig. 3:**
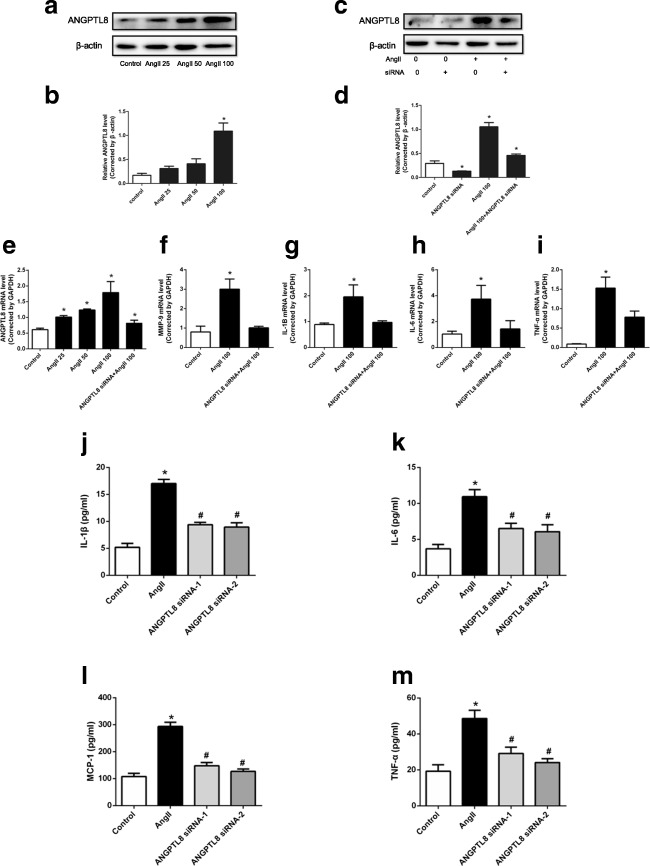
ANGPTL8 expression was increased in angiotensin II (AngII)-induced macrophage cells, while inhibition of ANGPTL8 via ANGPTL8 siRNA decreased the expression of ANGPTL8 and inflammatory factors. **a**, **b** ANGPTL8 levels in RAW264.7 cells were increased after AngII treatment, as assessed by Western blot. **c**, **d** ANGPTL8 siRNA decreased the expression of ANGPTL8 in RAW264.7 cells induced by AngII. **e** RAW264.7 cells were treated with 25, 50, or 100 nmol/L AngII for 24 h. The 100 nmol/L AngII was used for the ANGPTL8 siRNA study. **f**–**i** qPCR analysis of gene expression of ANGPTL8 and the inflammatory factors TNF-α, IL-6, MMP-9, and IL-1β in RAW264.7 cells. **j**–**m** ELISA analysis of macrophage secreted IL-1β, IL-6, TNF-α, and MCP-1. The RAW264.7 cells were treated with AngII (100 nmol/L) except for the control group. ANGPTL8 siRNA-1 and ANGPTL8 siRNA-2 represent the two sequences of ANGPTL8 siRNA. *P < 0.05 vs. control group, ^#^*P* < 0.05 vs. AngII group. ANGPTL8 siRNA decreased the expression of the inflammatory factors induced by AngII in RAW264.7 cells. Data are presented as mean ± SEM. *P < 0.05

Western blot analysis showed that AngII administration promoted ANGPTL8 expression in macrophage cells in a concentration-dependent manner (Fig. 3a, b), while ANGPTL8 siRNA inhibited the effect of AngII on ANGPTL8 induction (Fig. 3c, d). A similar trend was observed for ANGPTL8, IL-6, IL-1β, TNF-α, and MMP-9 mRNA levels after AngII treatment in macrophages. ANGPTL8 siRNA decreased the mRNA expression of ANGPTL8, IL-6, IL-1β, TNF-α, and MMP-9 in macrophages (Fig. 3e–i). In addition, we detected cytokines and chemokines secreted by macrophages using ELISA and found that AngII treatment could increase IL-1β, IL-6, TNF-α, and MCP-1 secretion. Furthermore, ANGPTL8 siRNA decreased the AngII-induced expression of cytokines and chemokines detected in the macrophage culture media (Fig. 3j–m).

### Effect of ANGPTL8 on HUASMC Apoptosis

**Fig. 4:**
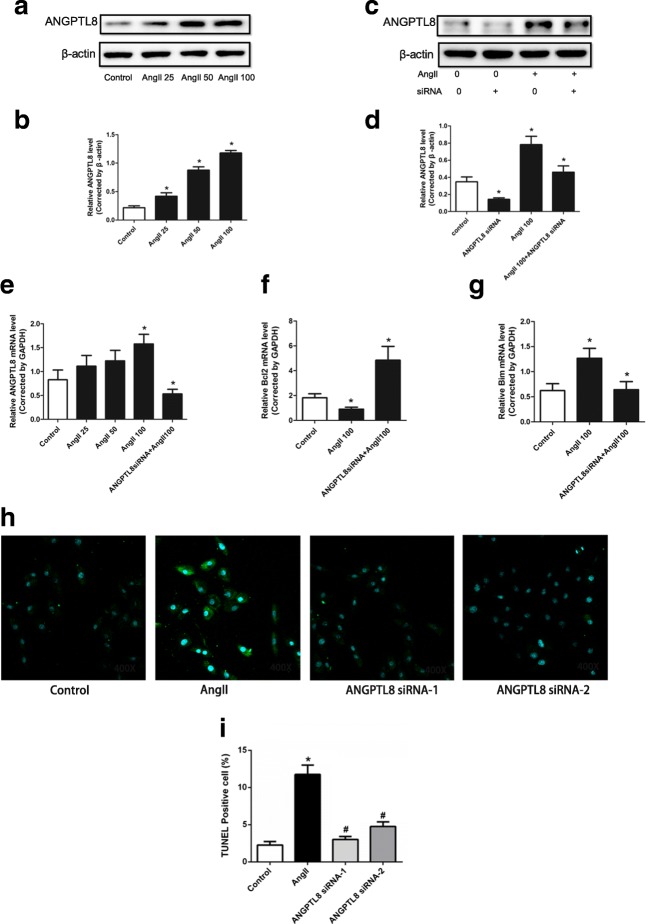
ANGPTL8 expression was increased in angiotensin II (AngII)-induced HUASMCs, while inhibition of ANGPTL8 via ANGPTL8 siRNA decreased the expression of ANGPTL8 and apoptosis markers. **a**, **b** ANGPTL8 levels in HUASMCs were increased after AngII treatment, as assessed by Western blot. **c**, **d** ANGPTL8 siRNA decreased the expression of ANGPTL8 in HUASMCs induced by AngII. **e** HUASMCs were treated with 25, 50, or 100 nmol/L AngII for 24 h. The 100 nmol/L AngII was used for the ANGPTL8 siRNA study. **f**, **g** qPCR analysis of the gene expression of ANGPTL8 and the antiapoptotic genes Bcl-2 and Bim in HUASMCs. **h**, **i** TUNEL staining to detect the apoptosis of HUASMCs induced by AngII. The HUASMCs were treated with AngII (100 nmol/L) except for the control group. ANGPTL8 siRNA-1 and ANGPTL8 siRNA-2 represent the two sequences of ANGPTL8 siRNA *P < 0.05 vs. control group, ^#^*P* < 0.05 vs. AngII group. ANGPTL8 siRNA decreased the expression of the apoptotic factors and decreased the apoptosis of HUASMCs induced by AngII. Data are presented as mean ± SEM. *P < 0.05

Western blot analysis showed that AngII administration also promoted ANGPTL8 expression in HUASMCs in a concentration-dependent manner (Fig. 4a, b), while ANGPTL8 siRNA inhibited the effect of AngII on ANGPTL8 induction (Fig. 4c, d). AngII administration also promoted ANGPTL8 mRNA expression in HUASMCs (Fig. 4e). Further, ANGPTL8 siRNA increased Bcl-2 mRNA levels, but reduced Bim mRNA levels, in HUASMCs (Fig. 4f, g). In addition, we detected the number of apoptotic HUASMCs via TUNEL staining and found that AngII administration increased the apoptosis of HUASMCs. Furthermore, ANGPTL8 siRNA decreased the AngII-induced apoptosis of HUASMCs (Fig. 4h, i).

## Discussion

In this study, we found that circulating ANGPTL8 levels in Chinese TAD patients were significantly elevated compared with controls. Our results also showed that ANGPTL8 was independently associated with the occurrence of TAD, while increased ANGPTL8 levels were positively correlated with arterial diameter and hs-CRP, after adjusting for confounding factors in the study population. ANGPTL8 combined with hs-CRP and D-dimer may be a predictor of TAD. We also found that AngII promoted the expression of ANGPTL8, accompanied by an increase in inflammatory factors, while inhibition of ANGPTL8 by ANGPTL8 siRNA reversed these effects. Together, these results highlight the potential role of ANGPTL8 in TAD and inflammatory pathways. Further, ANGPTL8 increased apoptosis and apoptosis-related mRNA levels in HUASMCs, while inhibition of ANGPTL8 by ANGPTL8 siRNA reversed these effects.

Many studies have reported roles for inflammation in the pathogenesis of TAD, as evidenced by the presence of inflammatory cells, especially neutrophils and macrophages [24]. hs-CRP, a marker of inflammation, can promote atherosclerosis and predict cardiovascular events [25]. It has been demonstrated that a patient’s initial hs-CRP level may be a simple and readily available biomarker that could help emergency care staff identify cardiovascular disease patients at high risk of cardiovascular events and who could benefit from inflammation modulation therapy to reduce cardiovascular risk [26]. In addition, there were time-dependent changes in plasma inflammatory biomarkers including hs-CRP and IL-6 in type A aortic dissection patients. The time to the peak level of hs-CRP was shorter and the duration of persistently high hs-CRP level was longer in the aortic dissection with complication patients [27]. Furthermore, we previously found that hs-CRP was associated with abdominal aortic aneurysms [28]. In the present study, serum hs-CRP levels were significantly increased in TAD patients compared with controls, which provides further support for increased inflammation in TAD patients.

ANGPTLs are widely expressed in many tissues including the liver, as well as in the vascular system, and these proteins play important roles in lipid metabolism, inflammation, and angiogenesis [29]. ANGPTL2 has been proven to play an important role in the damage of vascular endothelial cells and the promotion of macrophage infiltration in atheromatous plaques [30], and it has become a hotspot in cardiovascular disease research. ANGPTL4 was also involved in inflammation, and the production of pro-inflammatory cytokines including TNF-α, IL-1β, and IL-6 were decreased in ANGPTL4 knocked down mesangial cells. Inhibition of ANGPTL4 markedly suppressed the expression of extracellular matrix (ECM) proteins, collagen IV, and fibronectin, in high glucose-stimulated mesangial cells [31]. Previous studies found a positive association between ANGPTL8 expression and degenerative grades of intervertebral disc degeneration. Silencing ANGPTL8 attenuated the degradation of the anabolic protein type collagen II, and IL-6 through inhibition of NF-κB signaling activation [32]. This study suggests that ANGPTL8 plays an important role in the degradation of ECM and inflammation regulation. The pathophysiology of TAD is characterized by changes in the ECM and inflammation; however, the role of ANGPTL8 on TAD has not been reported. In the present study, serum ANGPTL8 levels were independently associated with serum hs-CRP in TAD patients. Our in vitro results showed that ANGPTL8 was increased in AngII-induced macrophages, accompanied by increased IL-6, IL-1β, TNF-α, and MMP9 mRNA expression in macrophages, while knockdown of ANGPTL8 decreased inflammation in AngII-induced macrophages. In addition, AngII treatment could increase macrophage-secreted IL-1β, IL-6, TNF-α, and MCP-1, and ANGPTL8 siRNA decreased AngII-induced expression of cytokines and chemokines in macrophage medium. Our results demonstrated that ANGPTL8 might participate in the development of TAD by promoting inflammation.

VSMC apoptosis was also an important mechanism for TAD development with the Bcl-2 family playing an important role in VSMC apoptosis in TAD [33]. Previous studies employed ultrasound-targeted microbubble destruction to deliver the human ANGPTL8 gene to the rats with adriamycin cardiomyopathy. ANGPTL8 stimulated the proliferation of cardiac progenitor cells located at the epicardium [34], indicating ANGPTL8 might regulate cell proliferation. However, our in vitro study found that AngII decreased Bcl-2 mRNA levels in HUASMCs and increased Bim mRNA levels, accompanied with increased expression of ANGPTL8; treatment with ANGPTL8 siRNA reversed these effects. In addition, TUNEL staining showed that ANGPTL8 siRNA reduced the apoptosis of HUASMCs induced by AngII. Our results indicate that ANGPTL8 may change during the development of TAD via increased apoptosis of HUASMCs.

In the past decades, D-dimer and hs-CRP focused on evaluating acute TAD prognosis. However, D-dimer specificity was low when patients presented with false lumen thrombosis, less extensive disease, and younger age [35], and hs-CRP was easily influenced by acute infection, trauma, and bleeding [36]. Therefore, a novel biomarker with superior performance for predicting TAD is required. Recently, a combination of different biomarkers was proposed to improve the accuracy of predicting disease. For example, Giachino et al. reported that a combination of MMP-8 and D-dimer can increase the AUC of ROC curve of predicting acute aortic dissection [37]. In the recent study, a combination of tenascin-C and D-dimer or CRP can improve the performance of predicting in-hospital death from acute aortic dissection [38]. Proietta et al. reported MMP-12 as a new marker of Stanford-A acute aortic dissection, and MMP-12 was significantly increased in TAD patients [39]. Our results found that compared with ANGPTL8 alone, the AUC combination of ANGPTL8, hs-CRP, and D-dimer was up to 0.927, and the sensitivity and specificity were 98.46% and 79.49%, respectively. These data demonstrated that ANGPTL8 can enhance D-dimer’s ability to evaluate presence of TAD. In addition, according to the guidelines for TAD therapy, the diameter of the aorta at > 50 mm was associated with high rupture risk and required surgery [40]. After dividing the TAD cases into low rupture risk group (diameter < 50 mm) and high rupture risk (diameter > 50 mm), ANGPTL8 was associated with higher rupture risk of TAD. This result might be helpful for the clinical diagnosis and therapy of TAD in the future.

The ANGPTL8 protein can control triglyceride levels via regulation of LPL [41], so circulating ANGPTL8 levels were positively associated with serum TG levels. Thus, TG levels were adjusted in logistic regression in this study. ANGPTL8 was also reported to be negatively associated with HDL-C levels [42], while multiple logistic regression analysis showed that ANGPTL8 was associated with TAD after adjusting for HDL-C. Numerous studies have demonstrated that ANGPTL8 levels are positively correlated with FPG and insulin resistance [43]. However, we found no differences in FPG levels between TAD patients and controls, and no association of ANGPTL8 levels with FPG, in our subjects. Multiple logistic regression analysis found that ANGPTL8 was associated with TAD after adjusting for FPG. ANGPTL8 was shown to be an independent biomarker for hypertension, and the incidence of hypertension in TAD patients is very high. However, we found no effect of hypertension and no effect of antihypertension drugs on serum ANGPTL8 levels in TAD patients. Multiple logistic regression analysis found that ANGPTL8 was associated with TAD after adjusting for SBP and DBP. Several case-control studies have documented Hcy elevation in patients with abdominal aortic aneurysms [44]. Furthermore, there is a correlation between Hcy levels and aneurysm size as well as high Hcy levels and aneurysm expansion [45]. Hcy is also increased in Marfan patients with severe cardiovascular manifestations, in particular aortic dissection, compared with Marfans with mild cardiovascular disease [46]. Hcy is a significant risk factor for hypertension [47], and hypertension has a high prevalence in TAD patients [48]. Further, ANGPTL8 plasma levels are increased in hypertension patients [49]. Multiple logistic regression analysis found that ANGPTL8 was associated with TAD after adjusting for Hcy.

Care was taken to avoid bias in the present study. Evaluation of ANGPTL8 was performed according to the manufacturer’s instructions by a trained experimenter who was unaware of the patients’ clinical data. For the statistical analyses, adjustments were made for the confounding effects of risk factors for TAD and circulating ANGPTL8 levels. The sample size and statistical power of this explorative study were also sufficient (*P* = 0.802, *α* = 0.05, *P*_0_ = 0.5, *n* = 150, OR = 1.587). Nevertheless, this study has some limitations. First, as this was a case-control study, we can only show associations not causality. Furthermore, all study participants were Chinese. Thus, the findings may not be generalizable to other ethnicities. Our findings should be confirmed in other populations.

## Conclusions

Circulating ANGPTL8 levels were significantly higher in Chinese TAD patients compared with controls. Circulating full-length ANGPTL8 levels were an independent risk factor for TAD and were positively associated with diameter and hs-CRP. ANGPTL8 combined with hs-CRP concentration and D-dimer may be a useful predictor of TAD. The association of a combined higher ANGPTL8 and hs-CRP levels with a greater incidence of TAD needs to be verified in a larger sample size. Finally, increased ANGPTL8 was correlated with inflammation, indicating a higher inflammatory condition in TAD patients.

## Electronic Supplementary Material

ESM 1 (DOCX 377 kb)
