# Supplementary material for: Increased Circulating Angiopoietin-Like Protein 8 Levels Are Associated with Thoracic Aortic Dissection and Higher Inflammatory Conditions
Source: Cardiovasc Drugs Ther. 2020 Feb 7;34(1):65–77. doi: 10.1007/s10557-019-06924-7 (PMC7093348; doi:10.1007/s10557-019-06924-7)
Supplement: Supplementary file 1 — (DOCX 377 kb) [file 10557_2019_6924_MOESM1_ESM.docx]

Supplemental figure S1


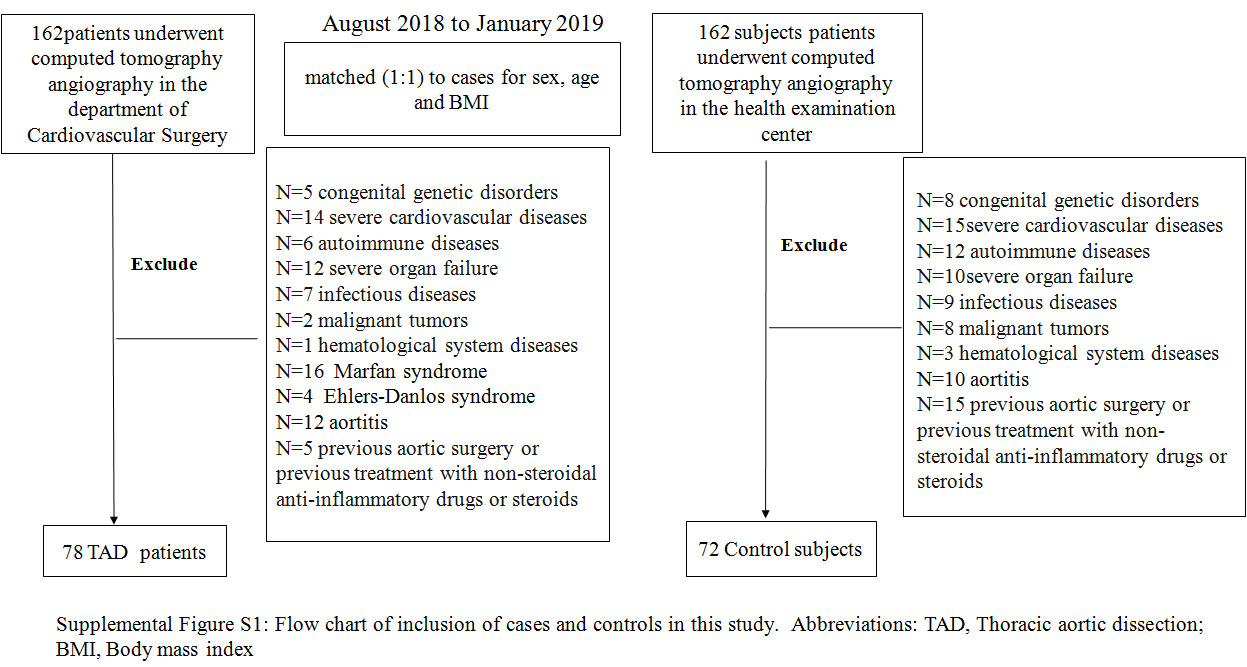


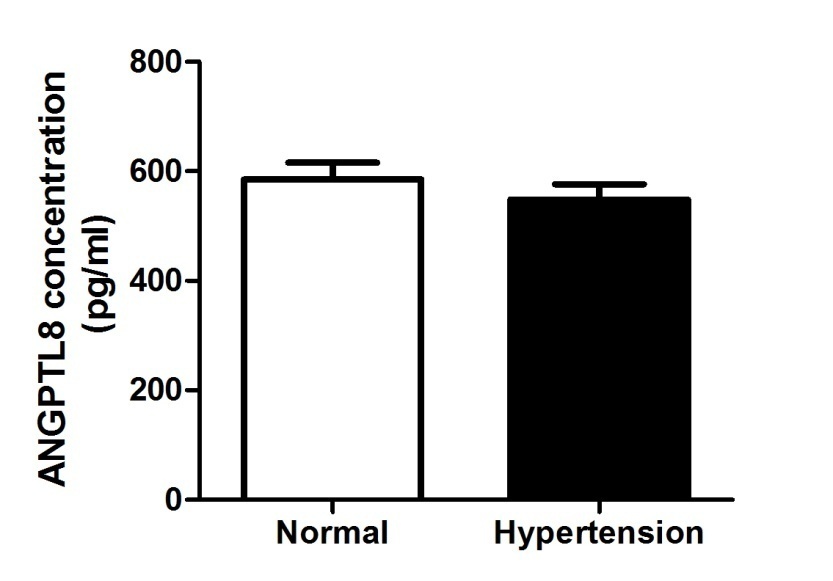


Supplemental firuge S2. Circulation level of ANGPTL8 in TAD patients with or without hypertension.

Plasma levels of ANGPTL8 in TAD subjects without hypertension vs. those with hypertension as measured by ELISA. * p < 0.05, as determined using Student’s t-test


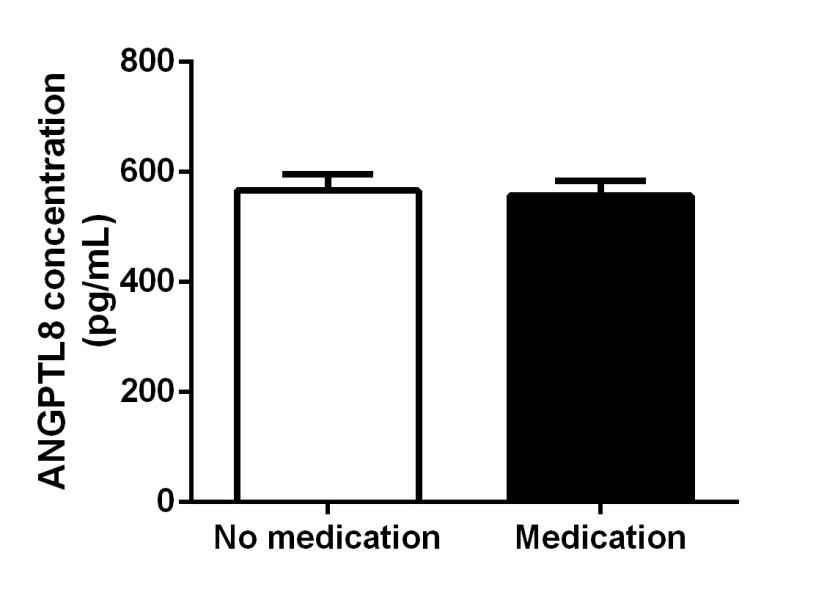


Supplemental Figure S3. Circulation level of ANGPTL 8 in hypertension patients with or without medication. Plasma levels of ANGPTL8 in TAD subjects without anti-hypertension medication vs. those with anti-hypertension medication as measured by ELISA. * p < 0.05, as determined using Student’s t-test
